# *Syzygium malaccense* leaves methanol extract modulate some biochemical and inflammatory markers and prostate histology of testosterone-estradiol valerate induced benign prostatic hyperplasia in rats

**DOI:** 10.22038/AJP.2023.23526

**Published:** 2024

**Authors:** Ngozi Kalu Achi, Chinedum Ogbonnaya Eleazu, Chimaraoke Onyeabo, Winner Kalu, Kate Eleazu

**Affiliations:** 1 *Department of Biochemistry, Michael Okpara University of Agriculture, Umudike, Umuahia, Abia State, Nigeria*; 2 *Department of Biochemistry, Alex Ekwueme Federal University, Ndufu-Alike, Ebonyi State, Nigeria*; 3 *Department of Biochemistry, Rhema University, Aba, Abia State, Nigeria*; 4 *Department of Biochemistry, Ebonyi State University, Ebonyi State, Abakaliki, Nigeria*

**Keywords:** Prostate hyperplasia, Nutrition, Oxidative stress, Antioxidants, Toxicology

## Abstract

**Objective:**

The effect of *Syzygium malaccense* methanol leaf extract (SMLE) on some parameters of testosterone-estradiol valerate induced benign prostatic hyperplasia (BPH) in rats was assayed.

**Materials and Methods:**

Thirty male albino rats were used and they were grouped as: Control: received 1 mL/kg olive oil (oral and subcutaneous); BPH: received subcutaneously 9 mg/kg dihydrotestosterone (DHT)+0.9 mg/kg estradiol valerate (ESV) and orally 1 ml/kg olive oil; finasteride: received 9 mg/kg of DHT+0.9 mg/kg ESV (subcutaneously) and 5 mg/kg finasteride (orally) and test groups 1 and 2: received 9 mg/kg of DHT+0.9 mg/kg ESV (subcutaneously) and 200 and 400 mg/kg SMLE (orally). The duration of the treatment was 28 days.

**Results:**

The BPH group had increased prostatic total proteins, oxidative stress, interleukin 8, tumor necrosis factor-α, prostate weights, serum concentrations of prostate specific antigen, estradiol, follicle stimulating hormone, and C-reactive protein, dyslipidaemia, altered prostate histology and hormonal levels but had no significant change (p>0.05) in haematological indices relative to the control. Finasteride or *S*.* malaccense* modulated most of these parameters as corroborated by prostate histology. Acute toxicity study indicated the non-toxicity of SMLE. SMLE showed strong *in vitro* antioxidant activity which corroborated its *in vivo* antioxidant activity.

**Conclusion:**

The study showed that *S*.* malaccense *could be useful in the management of BPH

## Introduction

Benign prostatic hyperplasia (BPH) is a non-malignant enlargement of the prostate gland (Kalu et al., 2016a; Kalu et al., 2016b). It is the most common urological condition that affects about fifty percent of men who are above the age of 60 years. Given the gradual increase in the population of the elderly, the number of BPH patients is expected to increase further (Choi et al., 2019). In terms of the etiology of BPH, several factors have been reported including: inflammatory mediators, growth factors, sex hormones, dietary factors, neurotransmitters, or environmental factors and oxidative stress (Eleazu et al., 2017; Zhang et al., 2020).

In its early stage, BPH causes enlargement of the prostate, compressing the bladder, resulting to lower urinary tract symptoms (LUTS) such as: urgency, frequency, straining, urinary intermittency, weak stream, incomplete bladder emptying, and nocturia. As the enlargement of the prostate progresses, it could result to more life-threatening complications such as: loss of bladder function, renal failure and even death (Kalu et al., 2016a and b; Choi et al., 2019; Jeon et al., 2017).

The two major medications that are currently being used to treat BPH include the α1-adrenergic receptor antagonists (doxazosin, terazosin, and tamsulosin) and the 5α-reductase inhibitors (e.g. finasteride). While these conventional drugs have been found to be effective in BPH treatment, the side effects that have been associated with their usage, have led to increased search for alternative means of managing or treating this disease (Eleazu et al., 2017).

*Syzygium malaccense* (L.) (synonym- *Eugenia malaccensis* L.), popularly known as ‘Malay apple’ or ‘Mountain apple’, is a berry that belongs to the Myrtaceae family and has its origin from Malaysia and India, but is widespread throughout the tropical regions (Savitha et al., 2011; Batista et al., 2017). The fruits are eaten fresh or in the form of handmade products (Batista et al., 2017). The leaves are also edible as they are eaten raw with rice or are cooked and eaten as vegetable (Morton, 1987; French, 2004). Different parts of this plant, such as the seeds, bark, fruit, and leaves are used traditionally as anti-inflammatory, antiviral, antifungal, antibacterial, antibiotic and anti-edema agents as well as a remedy for itching and as diuretics (Savitha et al., 2011; Locher et al., 1995). The *in vitro* anti-diabetic property of the leaves has been reported (Arumugam et al., 2014) and the study of hydro distilled essential oil from the fresh leaves of *S. malaccense* grown in Nigeria, showed the oil to be largely composed of monoterpenes (61.1%) and sesquiterpenes (30.8%) (Karioti et al., 2007). Although previous studies (Savitha et al., 2011; Batista et al., 2017; Arumugam et al., 2014) showed that the leaves of *S. malaccense* exhibited strong *in vitro *antioxidant activity, there is paucity of such information in the literature on *S. malaccense* leaves that are grown in Nigeria. Given the traditional usage of the leaves of this plant as anti-inflammatory agent and its reported *in vitro* antioxidant properties (Batista et al., 2017; Arumugam et al., 2014) and considering that berries were reported to possess antitumoral properties (Batista et al., 2017), we hypothesized that this plant could be of importance in the management of BPH.

As a way of testing our hypothesis, we determined the effect of *S. malaccense* methanol leaf extract on some biochemical, inflammatory and histological indices in BPH rats induced with testosterone-estradiol valerate.

## Materials and Methods

**Reagents and chemicals**

The reagents/chemicals such as: Riboflavin, Nitrotetrazolium Blue chloride, L-Glutathione (reduced), sodium thiosulphate, sodium benzoate, sodium azide, sodium nitroprusside, curcumin, dihydrotestosterone (DHT) and estradiol valerate (ESV), which we used for this study were purchased from Sigma Aldrich Chemical Company, UK. Other reagents/chemicals that were used for this study but which we did not list here, were also of analytical grade. 

**Collection and identification of plant materials**

Fresh mature leaves of *S. malaccense *were collected from Ezinihitte Mbaise, Imo State, Nigeria. The leaves were identified by Mr Ibe Ndukwe, a Taxanomist in the herbarium section of the Department of Forestry and Environmental Management, Michael Okpara University of Agriculture, Umudike (MOUAU), Abia State, Nigeria. A voucher specimen was kept in the herbarium of the same Department for future reference. 

**Preparation and extraction of plant material**

The leaves were washed with clean water and thereafter, they were air dried at room temperature (25-27^o^C) for 48 hr. The dried leaves were pulverised into fine powder using a manual grinding machine (model Corona Ref, 121, landers). Certain quantity (150 g) of the powdered sample was weighed and dissolved in 1 L of 95% methanol, stirred and kept for 48 hr. Thereafter, it was filtered using a muslin cloth. The filtrate was heated at 40^o^C using a water bath until all the methanol was removed, to get the dried crude extract. The extract was subsequently weighed and used for acute toxicity, *in vitro* antioxidant activity and experimental BPH studies. The yield of the extract (in percentage) was calculated as: [Weight of the extract/Weight of the powdered sample] x 100 (Achi et al., 2017). The weight of the *S. malaccense* extract was 18.76 g while the percentage yield was 12.51%. 

**Animal experiment**

Thirty male Wistar rats (aged 8 weeks old) and adult mice of both sexes (20±5 g) were procured from the animal house of the Faculty of Biological Sciences, University of Nigeria Nsukka, Enugu State, Nigeria. The mice were used for studies on acute toxicity while the rats were used for BPH studies following two weeks of acclimatization to their environment, diets (Vital feed, Jos, Nigeria) and water which they had access to *ad libithum*. All animals that were used in this study were housed at 25^o^C in stainless cages under normal daylight/dark cycle and humid tropical conditions. The animal studies were carried out following ethical approval by the College of Veterinary Medicine, MOUAU, Abia State, Nigeria. The approval conformed to the guideline for animal care and handling that was given by the National Research Council (1985).

**Acute toxicity study**

The method of Lorke (1983) was used for acute toxicity testing in mice following their acclimatization. At the highest tested dose of 5000 mg/kg, no mortality or obvious signs of toxicity was observed. The lethal dose of the extract was therefore obtained as >5000 mg/kg. The acute toxicity study thus indicated the non-toxicity of SMLE

On the basis of the acute toxicity study and following conversion to rats’ equivalent dose (Freireich et al. 1966), the doses of 100 and 200 mg/kg were selected for BPH studies (Kalu et al., 2016 a and b). 

**BPH studies**

Following two weeks of acclimation of the rats, they were randomized into five groups of six rats each and treated as shown in Table 1. Induction of BPH in the rats was carried out using a combination of DHT and ESV (in the ratio of 10:1) (Arumugam et al., 2014; Jeyaraj et al., 2000). The dose that was used for BPH induction was 9 mg/kg of DHT and 0.9 mg/kg of ESV and these were given by subcutaneous injection to the rats, every other day for 28 days (Kalu et al., 2016a). Finasteride and the extract were orally given, once daily for 28 days.

**Table 1:** Study groups and treatments

**Groups**	**Groups**	**Subcutaneous injection**	**Oral administration**
Group 1	Control	1 mL/kg (olive oil)	1 mL/kg olive oil
Group 2	BPH	9 mg/kg DHT + 0.9 mg/kg ESV	1 mL/kg olive oil
Group 3	Finasteride	9 mg/kg DHT + 0.9 mg/kg ESV	5 mg/kg finasteride
Group 4	Test group 1	9 mg/kg DHT + 0.9 mg/kg ESV	100 mg/kg *S. malaccense*
Group 5	Test group 2	9 mg/kg DHT + 0.9 mg/kg ESV	200 mg/kg *S. malaccense*

DHT- Dihydrotestosterone; ESV-Estradiol valerate; BPH- Benign Prostatic Hyperplasia

**Experimental procedure**

The body weights of the animals were recorded weekly. At the end of 28 days, the animals were fasted overnight and the next day, blood was collected from their heart into EDTA tubes for haematological assays while the rest of their blood samples was collected into plain tubes. Serum was obtained from each blood sample following centrifugation at 3000 x g for 10 min and the obtained sera were analyzed for prostate specific antigen (PSA), testosterone, DHT, follicle stimulating hormone (FSH), estradiol, C-reactive protein, triacylglycerol (TAG), total cholesterol, High Density Lipid Cholesterol (HDL), Low Density Lipid Cholesterol (LDL) and Very Low-Density Lipid Cholesterol (VLDL). 

The prostates of the rats were removed, blotted and weighed. The relative weights of the prostates were calculated and are reported as g/1000 g. After weighing the prostates, the ventral lobes were divided into two. One half was fixed in 10% formalin for histology (Cai et al., 2018). The other half was homogenized in ice-cold tris (hydroxymethyl) aminomethane buffer (pH 7.4) and the homogenate was analyzed for total proteins, antioxidant status- reduced glutathione (GSH), catalase (CAT), superoxide dismutase (SOD), glutathione peroxidase (GPx), lipid peroxidation marker-malondialdehyde (MDA) and inflammatory markers- interleukin 8 (IL-8) and tumor necrosis factor (TNF-α). The results of the prostatic antioxidant status, lipid peroxidation and inflammatory markers were normalized with the prostatic protein contents. 

**Determination of hormonal profile in the sera**

The testosterone, DHT, estradiol and FSH concentrations in the sera of the rats were assayed by using the Rats’ testosterone, DHT, estradiol (E2) and FSH ELISA Kits (Cusabio, USA) (Babiel et al., 1990; Bouve et al., 1992; Shin et al., 2012) following the instructions that were given by the manufacturers. Results are reported as ng/mL for testosterone, pg/mL for DHT and estradiol, and mlU/mL for FSH. 

**Assay for inflammatory markers levels in the sera and prostate**

Assay for C-reactive protein concentrations in the sera of the rats was carried out using Rat C-Reactive Protein ELISA Kit (Cusabio, USA) following the instructions of the manufacturer. Results that were obtained are reported as ng/mL. Assay of IL-8 concentrations in the prostate of the rats was carried out using Rat Interleukin 8 ELISA kit (Cusabio, USA) following the instructions of the manufacturer. Results are reported as pg/mg protein. TNF-*α* concentrations in the prostates of the rats were determined using Rat Tumor Necrosis Factor Alpha ELISA Kit (ELabscience, USA) following the instructions of the manufacturer. Data that were obtained are expressed as pg/mg of protein. 

**Assay for serum PSA and total prostate proteins**

Serum concentrations of PSA were determined using the Rat Prostate Specific Antigen (PSA) ELISA Kit – Cusabio following the instructions of the manufacturer (Stowell et al., 1991) and results are expressed as pg/mL. The total protein concentrations in the rats’ prostates were determined using the method of Tietz (1995). 

**Assay for prostate antioxidant status and lipid peroxidation markers**

GSH concentrations in the rats’ prostates were measured following the guideline of Annuk et al. (2001) and results are reported as nmol GSH equivalent/mg protein. CAT activity was determined using the method of Chandran et al. (2014) and results obtained are reported as units/mg of protein. SOD activity was determined using the Nitro Tetrazolium Blue reduction method (Al Batran et al., 2013). The activity of SOD is reported as units/mg of protein. Glutathione peroxidase activity was determined using the method of Dogan et al. (1994). The activity of GPx is reported as units/mg of protein. MDA (marker of lipid peroxidation) was measured using the protocol of Chatterjee et al. (2000) as previously described (Eleazu et al., 2020) and results are reported as nmol of MDA per mg protein. 

**Determination of lipid profile in the sera**

Total cholesterol and HDL concentrations were determined following the method of NCEP (2001). The concentration of TAG was measured following the protocol of Tietz (1995). LDL and VLDL concentrations were calculated following the protocol of Friedwald et al. (1972). 

**Assay for haematological parameters**

Packed cell volume (PCV), white blood cells (WBC), red blood cells (RBC), hemoglobin (Hb), mean corpuscular volume (MCV), mean cell haemoglobin (MCH) and mean cell haemoglobin concentration (MCHC) were determined using a hematology analyzer. 

**Prostate histology**

Sections of the fixed prostate tissues were dehydrated with alcohol, cleared with xylene and embedded in molten paraffin wax. On solidifying, the paraffin blocks were subsequently sectioned at 5 μm using a microtome. The sections were subsequently stained with hematoxylin and eosin (H&E) for viewing under a microscope (Cai et al., 2018; Lee et al., 2017). Photomicrographs of the tissues were taken with a light microscope at x100 magnification. 

***In vitro*** **antioxidant studies**

**Total antioxidant activity**

The total antioxidant activity of the extract was determined based on its scavenging activity on 2,2-azinobis (3-ethylbenzothiazoline-6-sulfonate radical (ABTS) following the method of Zheleva-Dimitrova et al. (2010). Here, stock solutions of ABTS (7 mM) and potassium persulfate (2.4 mmol) were prepared. Thereafter, working solutions were prepared by mixing 5 mL of the ABTS stock solution and equivalent volume of the potassium persulfate solution and the setup was left in the dark at room temperature for 14 hr. Prior to usage, dilution of the solution was done by mixing 1 mL of ABTS solution and 60 mL of methanol to get an absorbance of 0.700 ± 0.020 at 734 nm using a spectrophotometer. The extract (1 mL) was reacted with an equal volume of the ABTS solution and the absorbance was also read at 734 nm after 7 min. The ABTS scavenging activity of the extract is expressed as percentage inhibition and was calculated as follows: Percentage inhibition = [Absorbance of control – Absorbance of sample]/ [Absorbance of control] x 100.

Where the ABTS radical (in methanol) is the control while the ABTS solution mixed with the extract/standard is the standard. 

From the calibration curves of percentage inhibition of ABTS radical versus the concentration of the samples, the half maximal inhibitory capacity (IC_50_) values of the extract were calculated. Vitamin C was used as the standard for this assay. The IC_50_ value represents the concentration of the extract that inhibited 50% of ABTS radical.

**Hydroxyl peroxide radical scavenging activity**

The hydrogen peroxide scavenging activity of the extract was determined using previously described protocols (Gulçın et al., 2003; Nna et al., 2018). In this assay, 3.4 mL of the extract and standards prepared in 0.1 M phosphate buffer (pH 7.4) were added to a test tube containing 0.6 ml of H_2_O_2_ solution (40 mM) in 0.1 M phosphate buffer (pH 7.4). A blank solution containing only phosphate buffer was included. Following incubation of the mixture at room temperature for 10 min, the absorbance was determined at 230 nm. The hydrogen peroxide radical scavenging activity is expressed in percentage and it was calculated as: [Absorbance of blank – Absorbance of sample]/ [Absorbance of sample] x 100. 

The antioxidant activity of the extract was determined from the calibration curve of the percentage inhibition of hydrogen peroxide radical versus the concentrations of the samples. From the calibration curves, the extracts’ IC_50_ values were calculated. Higher IC_50_ values indicate lower antioxidant activities. Vitamin C was used as the standard for this assay. 

**Nitric oxide radical scavenging assay**

The method of Boora et al. (2014) was used. A stock concentration of 10 mg/mL of the extract (in distilled water) was prepared and the extract was serially diluted with distilled water to make concentrations of 20 - 100 µg/mL. Similar concentrations (20 - 100 µg/mL) were also prepared for the standard. Thereafter, 0.5 mL of 10 mM sodium nitroprusside in phosphate buffered saline was added to 1 ml of the extract and the standard. The setup was then incubated at 25°C for 3 hr. To 5 mL of the incubated samples, 5 mL of Griess reagent (prepared by mixing equal volumes of 1% sulfanilamide in 2.5% phosphoric acid and 0.1% naphthyl ethylene diamine dihydrochloride in 2.5% phosphoric acid) (prepared immediately before use) was added and absorbance of the chromophore that was formed was measured at 546 nm. The control (lacking the extract but with equivalent volume of buffer) was prepared in the same way as the test samples. 

The percentage of nitric oxide radical scavenging activities of the extract and the standard were calculated using the following formula: 

% Nitric oxide scavenged = [Absorbance of control – Absorbance of test]/ [Absorbance of control] x 100. Where absorbance of test = absorbance in the presence of the extract or standard. From the calibration curves of percentage of inhibition of nitric oxide versus the concentration of the extract, the IC_50_ values of the extract were calculated. Curcumin was used as the standard for this assay.

**Statistical analysis**

Statistical analysis was carried out using the IBM statistical package for social sciences (SPSS) 23 software (Chicago, IL, USA). Data are reported as means±standard deviation. One-way analysis of variance (ANOVA) was performed, and *post hoc* multiple comparisons were conducted using Duncan multiple range test. Statistical significance was set at p<0.05. Graphs were generated using GraphPad Prism version 9.5.1 (GraphPad Software Inc., San Diego, CA, USA).

## Results

**Acute toxicity study**

**Figure 1:**
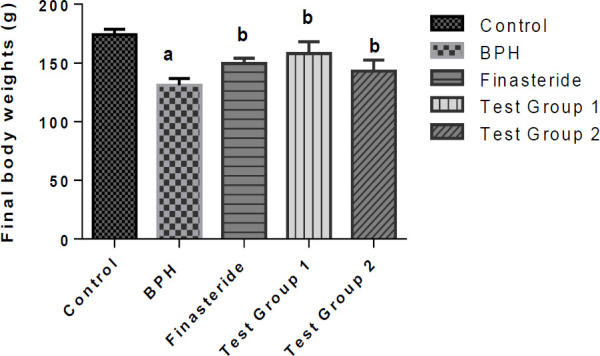
Final body weights of rats. Values are reported as means±SD. ^a^p<0.05 in comparison with the control; ^b^p<0.05 in comparison with the BPH group.

The LD_50_ of *S. malaccense *leaf methanol extract was 5000 mg/kg. The final weights of the rats are presented in Figure 1. The final body weight of the BPH group was significantly decreased (p<0.05) in comparison with the control. In contrast, the final body weights of the finasteride, and test groups 1 and 2 rats were significantly increased as compared to the BPH group.

**Relative prostate weights**

**Figure 2:**
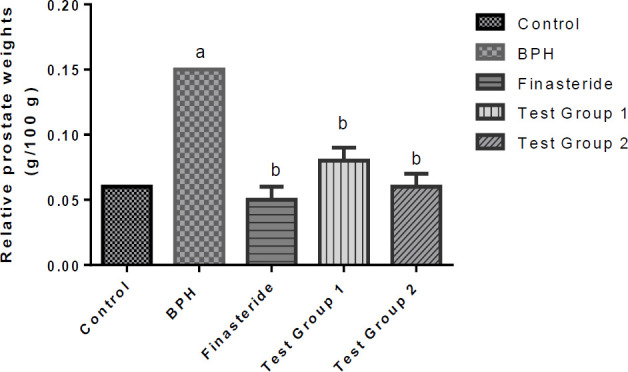
Relative prostate weights of rats. Values are reported as means ±SD. ^a^P<0.05 in comparison with the control; ^b^P<0.05 in comparison with the BPH group.

The relative prostate weights of the rats are shown in Figure 2. As shown in the figure, the relative prostate weight of the BPH group increased significantly (p<0.05) when compared to the control whereas the relative prostate weights of the finasteride, and test groups 1 and 2 rats were significantly decreased (p<0.05) as compared to the BPH group.

**Hormonal profile in the sera**

Table 1 shows the hormonal profile in the sera of the rats that were studied. As shown in the Table, the testosterone concentration of the BPH group was significantly decreased (p<0.05) in comparison with the control. In contrast, the testosterone concentrations of the finasteride, and test groups 1 and 2 rats were significantly increased (p<0.05) as compared to the BPH group. The DHT, FSH and estradiol concentrations of the BPH group increased significantly (p<0.05) compared to the control. On the contrary, the DHT, FSH and estradiol concentrations of the finasteride, and test groups 1 and 2 rats were significantly decreased (p<0.05) as compared to the BPH group. 

**Inflammatory markers levels in the sera and prostate**

Table 3 presents the inflammatory markers levels in the sera and prostates of the rats. As shown in the Table, the serum concentrations of C-reactive protein and the prostatic concentrations of IL-8 and TNF- α in the BPH group were significantly increased (p<0.05) in comparison with the control. On the contrary, the IL-8, TNF-α and C-reactive protein concentrations of the finasteride, and test groups 1 and 2 rats were significantly decreased (p<0.05) as compared to the BPH group. 

**Serum PSA and total prostatic proteins**

**Figure 3:**
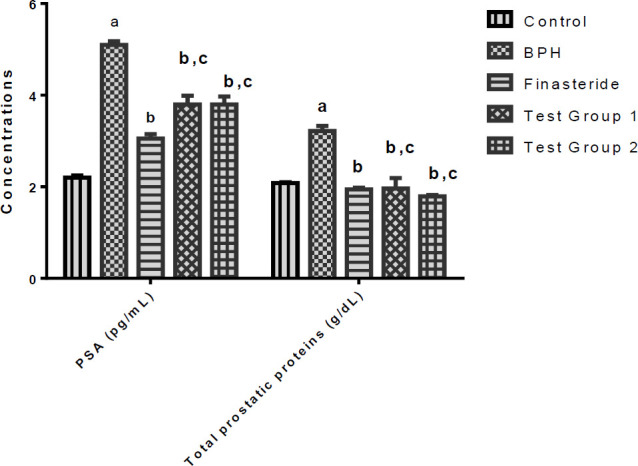
Serum PSA and total prostatic protein concentrations of rats. Values are reported as means±SD. ^a^p<0.05 in comparison with the control and ^b^p<0.05 in comparison with BPH. ^c^p>0.05 in comparison with the finasteride group. PSA- Prostate specific antigen

Figure 3 shows the serum PSA and total prostatic protein concentrations of the studied rats. The PSA concentration of the BPH group was significantly elevated (p<0.05) in comparison with the control whereas the PSA concentrations of the finasteride or test groups 1 and 2 rats decreased significantly (p<0.05) compared to the BPH group. Further, the PSA concentrations of the test groups were statistically similar to that of the finasteride group. 

Data presented in Figure 3 show that the total concentration of proteins in the prostate of the BPH group increased significantly (p<0.05) in comparison with the control. In contrast, the total prostatic protein concentrations of the finasteride or test groups 1 and 2 rats were significantly decreased (p<0.05) as compared to the BPH group. In addition, the total prostatic protein concentrations of the test groups were statistically similar to that of the finasteride group.

**Table 2:** Hormonal profile in the sera of rats

**Groups**	**Testosterone (ng/mL)**	**DHT (pg/mL)**	**Estradiol (pg/mL)**	**FSH (mlU/mL)**
Control	21 .18±1.56	25.97±1.36	252.31±0.62	3.15±1.77
BPH	13.32 ±1.21^a^	33.33±1.60^a^	377.71±0.25^a^	5.66±1.49^a^
Finasteride	19.13±1.81^b^	12.12±1.33^b^	233.40±1.23^b^	2.99±0.16^b^
Test group 1	19.50±1.37^b, c^	20.72±0.65^b^	261.15±1.21^b, c^	2.38±0.57^b,c^
Test group 2	19.98±1.43^b, c^	14.22±1.12^b,d^	249.21±1.26^b, c^	2.87±0.23^b,c^

Values are reported as means±SD. ^a^p<0.05 in comparison with the control; ^b^p<0.05 in comparison with the BPH, ^c^p>0.05 in comparison with the finasteride, and ^d^p<0.05 in comparison with the finasteride group. DHT- Dihydrotestosterone; FSH- Follicle stimulating hormone

**Table 3:** Inflammatory markers levels in the sera and prostates of rats

**Groups**	**IL-8 (pg/mg protein)**	**TNF- α ** **(pg/mg protein)**	**C-reactive protein (ng/mL)**
Control	10.22±1.34	0.52±0.14	359.11±1.14
BPH	21.15±1.19^a^	1.91±0.02^a^	678.52±1.64^a^
Finasteride	16.85±0.18^b^	1.18±0.02^b^	566.11±1.47^b^
Test group 1	12.77±1.54^b^	1.10±0.01^b, c^	524.13±1.14^b^
Test group 2	11.55±1.17^ b^	0.93±0.29^ b, c^	412.15±1.22^b^

Values are reported as means±SD. ^a^p<0.05 in comparison with the control; ^b^p<0.05 in comparison with the BPH; and ^c^p>0.05 in comparison with the finasteride group. IL-8- Interleukin 8 (Prostate) TNF-α- Tumor necrosis factor alpha (prostate); C-reactive protein (sera)

**Prostate antioxidant status and lipid peroxidation marker**

Table 4 shows the antioxidant status and marker of lipid peroxidation (MDA) in the prostates of the studied rats. The concentration of GSH in the BPH group decreased significantly (p<0.05) in comparison with the control whereas the concentration of GSH in the finasteride and test groups 1 and 2 rats were significantly increased (p<0.05) as compared to the BPH group.

Data presented in Table 4 show that the serum activity of CAT in the BPH group decreased significantly (p<0.05) in comparison with the control. Whereas, the serum activity of CAT in the finasteride or test group 1 rats did not differ (p>0.05) from the BPH group, the serum CAT activity of test group 2 rats increased significantly (p<0.05) as compared to the rats in the BPH group. 

As shown in Table 4, the SOD and GPx activities of the BPH group decreased significantly (p<0.05) compared to the control whereas the SOD and GPx activities of the rats in the finasteride and test groups 1 and 2 increased significantly (p<0.05) compared to the rats in the BPH group.

The concentration of MDA in the BPH group was found to be significantly higher (p<0.05) than the control. In contrast, the MDA concentrations of the rats in the finasteride and test groups 1 and 2 rats were significantly decreased (p<0.05) compared to the BPH group.

**Lipid profile in the sera**

Table 5 shows the lipid profile in the sera of the rats that were studied. As shown in the Table, there were significant increases (p<0.05) in the total cholesterol, TAG, LDL and VLDL but significant decreases (p<0.05) in the HDL concentrations of the BPH group in comparison with the control. On the other hand, there were significant decreases (p<0.05) in the total cholesterol, TAG, LDL and VLDL but significant increases (p<0.05) in the HDL concentrations of the finasteride or test groups 1 and 2 rats as compared to the BPH group. 

**Table 4:** Oxidative stress indices levels in the prostates of rats

**Groups**	**GSH (nmol/mg protein)**	**SOD (µnits/mg protein)**	**GP**_X_ ** (µnits/mg** **protein)**	**CAT (units/mg protein) **		**MDA (nmol/mg protein) **
Control	9.12±0.51	38.15±1.22	3.21±0.15	21.43±1.22		0.30±1.73
BPH	3.61±0.41^a^	19.33±1.55^ a^	0.30±0.03^a^	13.47±0.39^ a^		10.46±0.33^a^
Finasteride	5.19±0.79^b^	26.41±0.25^ b^	0.95±0.17^b^	14.60±0.82		4.58±0.99^b^
Test group 1	4.13±0.39	25.17±0.94^ b, c^	1.18±0.61^b, d^	15.22±0.39		3.48±0.46^b^
Test group 2	5.80±0.51^ b, c^	32.16±0.86^ b, d^	1.99±0.36^b, d^	18.19±0.78^b, d^		4.92±0.41^b^

Values are reported as means ±SD. ^a^p<0.05 in comparison with the control, ^b^p<0.05 in comparison with the BPH, ^c^p>0.05 in comparison with the finasteride and ^d^p<0.05 in comparison with the finasteride group. GSH- reduced glutathione; SOD-superoxide dismutase; GPx-Glutathione peroxidase; CAT-Catalase; MDA-Malondialdehyde

**Table 5:** Lipid profile in the sera of rats

**Groups**	**Cholesterol (mmol/L)**	**TAG ** **(mmol/L)**	**HDL ** **(mmol/L)**	**LDL** **(mmol/L)**	**VLDL** **(mmol/L)**
Control	3.43±0.21	1.53±0.09	1.70±0.05	1.22±0.04	0.31±0.02
BPH	7.04±0.25^a^	2.84±0.09^a^	0.29±0.05^a^	4.77±0.16^a^	0.57±0.05^a^
Finasteride	3.77±0.37^b^	1.78±0.10^b^	1.85±0.05^b^	1.56±0.06^b^	0.36±0.04^b^
Test group 1	3.80±0.06^b^	1.93±0.12^b^	1.39±0.28^b^	2.04±0.06^b^	0.37±0.03^b^
Test group 2	2.29±0.22^b^	1.55±0.12^b^	1.75±0.13^b^	0.23±0.07^b^	0.31±0.05^b^

Values are expressed as means±SD. ^a^p<0.05 in comparison with the control and ^b^p<0.05 in comparison with the BPH group. TAG: Triacylglycerol: HDL: High density lipoprotein cholesterol. LDL: Low density lipoprotein cholesterol. VLDL: Very low-density lipoprotein cholesterol

**Table 6:** Haematological parameters in the whole blood of rats

**Groups**	**PCV**	**WBC**	**RBC**	**Hb**	**MCV**	**MCH**	**MCHC**
Control	46.00±2.73	11.50±2.00	8.80±0.09	16.73±0.40	52.60±6.50	19.10±1.80	36.50±2.80
BPH	39.20±5.72	15.30±0.80	6.70±0.00	14.9±0.10	53.20±6.22	22.35±1.30	38.50±4.00
Finasteride	41.00±1.41	11.70±0.10	7.40±1.20	14.90±1.00	57.30±12.20	20.70±3.80	36.40±3.60
Test group 1	39.70±0.50	13.20±2.60	7.20±0.02	15.04±1.80	52.30±0.06	20.70±2.20	38.20±1.30
Test group 2	37.20±5.80	11.90±2.10	5.97±0.50^a^	13.10±0.03^a^	63.40±14.00	22.10±1.63	36.70±2.40

Values are reported as means ±SD. ^a^p<0.05 in comparison with the control. PCV: Packed cell volume. WBC: white blood cells. RBC: Red blood cells. Hb: Hemoglobin. MCV: mean corpuscular volume. MCH: Mean cell haemoglobin. MCHC: Mean cell haemoglobin concentration

**Haematological parameters in the blood**

Table 6 shows the haematological parameters in the whole blood of the rats that were studied. As presented in the Table, there were no significant differences (p>0.05) in the PCV, WBC, RBC, Hb, MCV, MCH or MCHC concentrations in the whole blood of the BPH group in comparison with the control. Similarly, there were no significant differences (p>0.05) in the PCV, WBC, RBC, Hb, MCV, MCH or MCHC concentrations in the whole blood of the finasteride or test groups 1 and 2 rats as compared to the BPH group. 

**Prostate histology**

**Figure 4:**
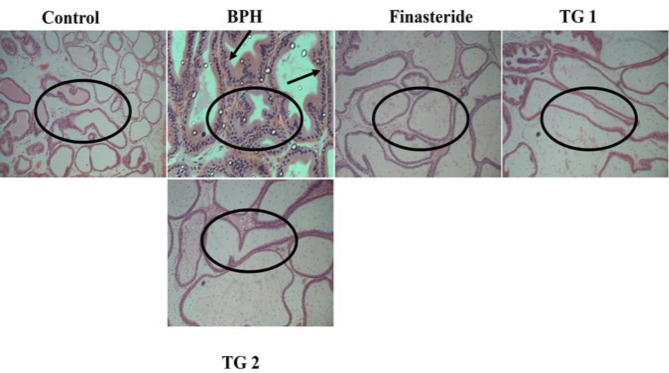
Histology of the prostates of rats. H&E staining. x 400 magnification. Histology of the control group showed normal acini cells with no structural alterations (encircled area). Histology of the BPH group showed evidence of several proliferating acini cells and inflammation of the acini cells (encircled area) with hyper secretion into the cells (black arrow). Histology of the finasteride group showed no proliferating cells (encircled area). Histology of the prostates of test group 1 showed very few proliferating acini cells with no inflammation of the cells (encircled area). Histology of the prostates of test group 2 showed no proliferating acini cells (encircled area). No inflammation was found in the acini cells of this group. There was also evidence of shrinkage of the prostate glands in this group (encircled area).

Figure 4 shows the histology of the prostates of the studied rats. As shown in Figure 4, histology of the prostate of the control group showed normal histological features of the prostate gland. There were normal acini cells (encircled area) with no structural alterations in this group.

Histology of the prostate of the BPH group showed altered prostate histology in comparison with the control, with evidence of several proliferating acini cells (encircled area), inflammation of the acini cells and hyper secretion into the cells (black arrows). The finasteride group prostate histology showed improved prostate histology with no proliferating acini cells (encircled area) in comparison with the BPH group. Histology of the prostate of test group 1 rats showed improved prostate histology in comparison with the BPH group with very few proliferating acini cells and no inflammation of the cells (encircled area). Histology of the prostate of test group 2 rats also showed improved prostate histology in comparison with the BPH group, with no proliferating acini cells and non-inflammation of the cells (encircled area). There was also evidence of shrinkage of the prostate glands in these test groups (encircled area). 

***In vitro* antioxidant studies**

**Figure 5a:**
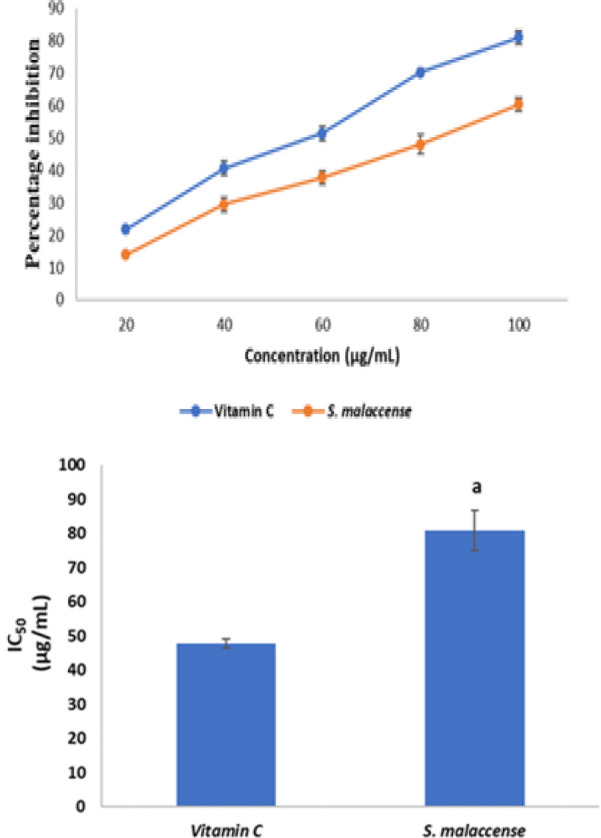
2,2-azinobis (3-ethylbenzothiazoline-6-sulfonate radical (ABTS) scavenging activity of Vitamin C and *S. malaccense. *Values are reported as means±SD. Figure 5b. IC_50_ values of vitamin C and *S. malaccense *for 2,2-azinobis (3-ethylbenzothiazoline-6-sulfonate radical (ABTS) scavenging activity. Values are reported as means±SD. ^a^p<0.05 in comparison with Vitamin C

Figure 5a shows the percentage inhibition of ABTS radical by *S. malaccense *leaf extract and vitamin C while Figure 5b shows the inhibitory capacity of the extract and vitamin C on ABTS radical. *S. malaccense *leaf extract had a strong inhibitory action on ABTS radical, producing an IC_50 _value of 80.87 µg/mL, which was higher (p<0.05) than that of vitamin C that had an IC_50_ value of 47.76 µg/mL. 

**Figure 6a:**
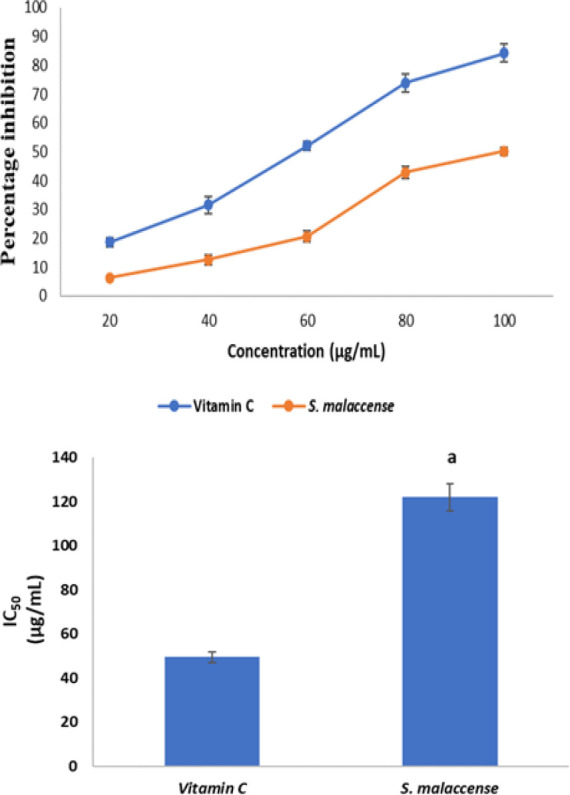
Hydrogen peroxide scavenging activity of vitamin C and *S. malaccense. *Values are reported as means±SD. Figure 6b. IC_50_ values of Vitamin C and *S. malaccense *for hydrogen peroxide scavenging activity. Values are reported as means±SD. ^a^p<0.05 in comparison with Vitamin C

Figure 6a shows the hydrogen peroxide radical scavenging activities of *S. malaccense* and vitamin C while Figure 6b shows their inhibitory capacities on hydrogen peroxide radical. *Syzygium malaccense *had a strong inhibitory action on hydrogen peroxide radical, producing an IC_50 _value of 122.05 µg/mL which was higher (p<0.05) than that of vitamin C that had an IC_50_ value of 49.51 µg/mL. 

**Figure 7a:**
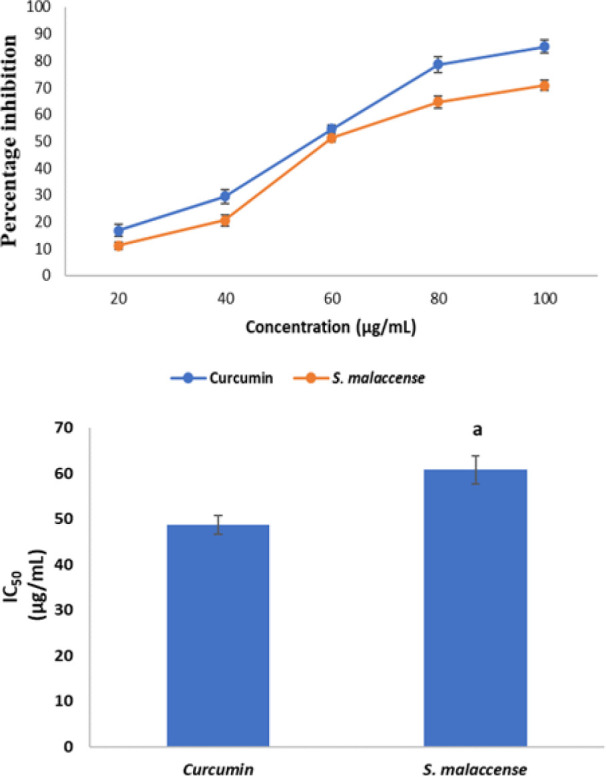
Nitric oxide scavenging activity of curcumin and *Syzygium malaccense. *Values are reported as means±SD. Figure 7b. IC_50_ values of curcumin and *S. malaccense *for nitric oxide scavenging activity. Values are reported as means±SD. ^a^p<0.05 in comparison with curcumin.

Figure 7a shows the nitric oxide radical scavenging activities of *S. malaccense* leaf extract and curcumin while Figure 7b shows their inhibitory capacities on nitric oxide radical. *Syzygium malaccense *had a strong inhibitory action on nitric oxide radical, producing an IC_50 _value of 60.78 µg/mL which was higher (p<0.05) than that of curcumin that had an IC_50_ value of 48.71 µg/mL. 

## Discussion

General behavior is a parameter of importance when assessing early toxicity signs (Ezeja et al., 2014; Emelike et al., 2020). The result of our study on acute toxicity which showed that the general behaviours of the mice were not affected by the extract, indicates the tolerance of the mice to the extract.

Based on previous reports (Kalu et al., 2016a; Emelike et al., 2020; Clarke and Clarke, 1977), substances with LD_50_ values ≥ 1000 mg/kg body weight could be considered to be safe for consumption. Therefore, the high LD_50_ of the *S. malaccense* leaf extract as obtained in this study might indicate its non-toxicity. 

Along with general behavior, weight changes are also considered to be essential in assessing early signs of toxicity (Emelike et al., 2020; Mbaka et al., 2017). At the end of the study, the BPH group was found to have decreased body weight compared to the control. Similar reports were also given by Kalu et al. (2016 a and b), Melvin et al. (2018), Eleazu et al. (2022) and Kim et al. (2017). This decrease has been attributed to loss of tissue proteins due to BPH insult (Kalu et al., 2016 a and b). Contrary to the loss of weight by the BPH group, the body weights of the finasteride, test groups 1 and 2 rats were increased which suggests mitigation of loss of tissue proteins by finasteride or the extracts due to the attenuation of BPH. 

Alteration of organ weights has indeed been reported as a sensitive index of measurement of the effect of toxicity of a drug (Kalu et al., 2016a). In this study, elevation in the relative prostate weight of the BPH group compared to the control was observed, which could be attributed to BPH-induced alterations in the prostate of this group (Kalu et al., 2016a; Lee et al., 2017). *Syzygium malaccense *demonstrated the ability to mitigate BPH-induced prostate enlargement as seen from the reduction in the relative prostate weights of test groups 1 and 2 rats, relative to the BPH group, and in a manner that was akin to finasteride (for test group 1).

The concentrations of the sex hormones (testosterone, DHT and estrogen) have been reported to affect the growth and malignant changes in the prostate gland (Kalu et al., 2016 a and b; Lee et al., 2017; Bartsch et al., 2000; Shibata et al., 2000). As men age, their testicular function declines and their androgen levels decrease because of increased conversion of testosterone to DHT in a reaction that is catalyzed by 5α-reductase (present in the prostate). DHT has higher affinity for androgen receptor (AR) than testosterone and because it dissociates slowly from the AR, it binds to more AR, enlarging the prostate gland (Eleazu et al., 2017; Lee et al., 2017). In addition to the conversion of testosterone to DHT, there is an increased conversion of androgen to estrogen (catalyzed by aromatase) in aging men, and the released estrogens cause increased expression of DHT and stimulation of the prostatic stroma, resulting in prostate proliferation and BPH (Eleazu et al., 2016a; Zhou et al., 2010; Kumar et al., 2010). 

As was obtained from this study, DHT administration increased the serum DHT concentration in the BPH group. We suspect that interaction of the exogenously administered DHT with the circulating DHT triggered this elevation. Our study further found that administration of exogenous DHT to the BPH group, led to a significant decline in their circulating testosterone concentrations. Similar reports were given by Page et al. (2011) in healthy men administered with exogenous DHT. In addition, the increased serum estradiol concentration in the BPH group, may not be unconnected with the exogenously administered estradiol valerate in the BPH group. 

The increased serum DHT and estradiol (an estrogen) concentrations in the BPH group, may have accounted for the enlargement of their prostates as seen in this study. Finasteride or test groups 1 and 2 rats had increased testosterone but decreased DHT and estradiol serum concentrations which may have played some role in the reduction of the enlarged prostates of these groups. Interestingly, the testosterone, DHT and estradiol modulating actions of *S. malaccense* in the BPH-challenged rats, at the doses used in this study, were akin to that of finasteride at 5 mg/kg*.*

Finasteride is an anti-BPH drug whose mechanism of anti-BPH action is known to be inhibition of 5α-reductase, attenuating the conversion of testosterone to DHT. Considering the side effects of finasteride, natural 5α-reductase inhibitors that can overcome these side effects are being sought for. Therefore, our study which showed increased testosterone but decreased DHT levels in the test groups 1 and 2 rats, suggests inhibition of 5α-reductase by *S. malaccense* at the doses that were used in this study. Current study therefore places spotlight on *S. malaccense* as a potential 5α-reductase inhibitor that could be of relevance in the treatment of BPH. 

The testes exert a negative feedback action on the hypothalamo-pituitary axis. This inhibits the synthesis of gonadotropin releasing hormone from the hypothalamus, thereby inhibiting the synthesis of the gonadotropins, one of which is FSH. Testosterone produced by the testes contributes to this feedback regulation. Therefore, increased testosterone levels are sensed by nerve cells in the hypothalamus, which decreases the release of gonadotrophin-releasing hormone and FSH (Tilbrook et al., 2001). The increased FSH concentrations in the BPH group could have arisen from decreased regulatory mechanism by testosterone due to its low concentrations in these groups of rats. In contrast, the decreased FSH concentration in the finasteride or test groups 1 and 2 rats suggests feedback inhibition of FSH by the increased testosterone concentrations of these groups of rats. 

Supporting studies have associated inflammatory infiltrates with the pathogenesis of BPH (Zhang et al., 2020; Kramer and Marberger, 2006; Vignozzi et al., 2012; Schalken, 2015). IL-8 is an inflammatory cytokine that has been reported to be the link between chronic prostate inflammation and BPH incidence. IL-8 is produced by the prostatic epithelial cells and its production induces the expression of fibroblast growth factor (FGF)-2, a stromal and epithelial growth factor that further promotes abnormal proliferation of the prostatic cells. As such, IL-8 is considered a key executor of stromal growth in BPH (Vignozzi et al., 2012; Gandaglia et al., 2013). Tumor necrosis factor-α is a pro-inflammatory cytokine that is mainly secreted by macrophages and it plays a vital role in initiating the inflammatory response and increasing oxidative stress in tissues (Eleazu et al., 2020). It has also been reported that the secretion of TNF-α, triggers the synthesis of IL-8 in different cells (Qazi et al., 2011). Hence, IL-8 and TNF-, are regarded as effective growth factors for the epithelial and stromal cells of the prostate (Cai et al., 2018). 

Our study found increased IL-8 and TNF-α concentrations in the prostatic tissue of the BPH group, suggesting chronic inflammatory condition in the prostates of these group of rats. 

Interestingly, the increased prostatic expression levels of IL-8 and TNF-α in the BPH group were attenuated in the finasteride or test groups 1 and 2 rats, suggesting the anti-inflammatory properties of finasteride or *S. malaccense*. Current finding therefore adduces a scientific rationale for the traditional usage of *S. malaccense *as an anti-inflammatory agent. 

C-reactive protein (CRP) is a major acute phase protein (APP) that participates in the acute phase response. CRP concentration increases shortly after a systemic inflammatory stimulus and as such, it is regarded as one of the earliest markers of a pathological condition (Khaki et al., 2016). Although the acute phase response (which lasts a few days) plays a positive role in the innate host defense mechanisms, increases in CRP have also been reported in chronic inflammation (Khaki et al., 2016). 

Going by the elevation in the prostate concentrations of IL-8 and TNF-*α *in the BPH group, the increased serum concentration of CRP in the BPH group may have been in response to chronic prostatic inflammatory condition in the BPH group. 

Similar to their effect on IL-8 and TNF-*α*, finasteride or *S. malaccense *demonstrated the ability to mitigate BPH-induced systemic inflammation by decreasing the serum CRP concentrations in the finasteride or test groups 1 and 2 rats. 

Current finding suggests the potentials of finasteride or *S. malaccense *to abrogate BPH-induced systemic inflammatory condition.

PSA is a glycoprotein that is produced through the phosphorylation of DHT within the stromal cells of the prostate and its serum concentration is reportedly high in patients with prostate cancer, BPH and prostatitis (Masrudin and Mohamad, 2015). In this study, we found marked elevations in the serum PSA concentrations of the BPH group. A reduction in serum PSA concentration has been associated with a reduction in prostatic hyperplasia due to the inhibition of prostatic 5α-reductase (Kalu et al., 2016a). Therefore, the decreased serum PSA concentrations in the finasteride and test groups 1 and 2 rats indicate the potentials of *S. malaccense* in the management of BPH. Interestingly, the PSA attenuating property of *S. malaccense* (at all doses used) was found to be similar to that of finasteride (at 5 mg/kg). 

Increased cell number has been correlated with an increase in protein levels (Ejike and Ezeanyika, 2011). The concentrations of total proteins in the prostates of the BPH group were markedly increased, suggesting that they may have played some role in the enlargement of their prostates. Similar reports on elevation of the prostatic protein levels in BPH rats were given by Kalu et al. (2016a). Our study further showed that *S. malaccense *administration attenuated the total protein concentrations in the prostates of the test group 1 and 2 rats to such an extent that they were similar to the total protein concentrations in the prostates of the finasteride group. We consider the decrease in the total protein concentrations in the prostates of the test group 1 and 2 rats to have contributed to the shrinkage of their prostatic tissues. 

Oxidative stress has been associated with the incidence and progression of prostatic hyperplasia especially in men who are aging (Eleazu et al., 2017). This is because during aging, there is an increased level of oxidative stress due to aberrant production of reactive oxygen species (Ishola et al., 2017). Additionally, the human prostate is reportedly vulnerable to oxidative DNA damage and this has been associated with its more rapid cell turnover and few DNA repair enzymes (Kalu et al., 2016b).

SOD catalyzes the dismutation of superoxide radical (which is obtained from the action of NADPH oxidase) into oxygen and H_2_O_2_. The released H_2_O_2 _is further broken down to H_2_O and O_2 _by GPx and in excess generation of H_2_O_2_, CAT is used to dismutate it to H_2_O and O_2_ (Eleazu et al., 2020; Voet et al., 2006). GSH is a tripeptide that is used to inactivate free radicals formed inside the red blood cells (Vasudevan et al., 2013).

As was seen in this present study, DHT/estradiol valerate model of BPH induction triggered peroxidation of lipids and oxidant stress in the prostate of the BPH group as evidenced from the decreased concentrations of SOD, GPx, CAT and GSH but increased MDA concentration in the prostate of the BPH group. 

On the other hand, while finasteride or test group 2 rats recorded remarkable increases in their GSH concentrations, the GSH concentration of test group 1 rats was mildly increased albeit not significant, suggesting the dose dependent action of *S. malaccense *in modulating the GSH concentration of the rats. As was also seen in this study, there were increased SOD and GPx activities but decreased MDA concentrations in the prostates of the finasteride or test groups 1 and 2 rats. Additionally, we observed dose dependent increases in the CAT activities of the test groups relative to the BPH and finasteride groups. These actions of finasteride or the test groups on the antioxidant markers and MDA in the prostates of the rats, suggest the potentials of finasteride or *Syzygium malaccense* to mitigate BPH induced oxidative stress in the prostate tissue. 

The non-significant change in the CAT activity of the finasteride group suggests its non-participation in the dismutation of the released H_2_O_2 _in the prostates of the rats (Eleazu et al., 2020).

Dyslipidemia refers to an elevation in blood concentrations of total cholesterol or LDL or decreased concentrations of HDL (Fodor, 2011). Dyslipidemia has been associated with the pathogenesis and progression of BPH (Gacci et al., 2017) and this informed our assay on the effect of BPH induction on the rats’ lipid profiles and the actions of finasteride or* Syzigium malaccense* leaf extract on their lipid profiles. 

In this study, we found increased concentrations of cholesterol, TAG, LDL, VDL but decreased HDL concentration in the sera of the BPH group, establishing the development of dyslipidaemia in this group of rats. Although how dyslipidaemia leads to BPH has not been fully elucidated, studies have associated decreased HDL and elevated TAG levels with prostate inflammation which can ultimately lead to BPH (Gacci et al., 2017). Our findings on the elevated serum concentrations of total cholesterol and LDL but decreased concentration of HDL in the BPH group, corroborate earlier studies of Nandeesha et al. (2006) on the elevation of these parameters in BPH. Further, the decreased concentration of HDL in the BPH group together with their increased total cholesterol and C-reactive protein concentrations suggest that they were at risk of developing coronary heart disease (Khandelwal and Sharma, 2017). Attenuation of TAG, total cholesterol, LDL, VLDL, C-reactive protein with corresponding increase in HDL in the finasteride or test groups 1 and 2 rats suggest the capacity of finasteride or *S. malaccense *to ameliorate dyslipidaemia*.*

The hematopoietic system serves as a target for toxic compounds including toxic plant extracts (Emelike et al., 2020). Therefore, assay of the haematological profile of the rats was done to further investigate the toxicity of *S. malaccense* leaf extract on the animals. 

As seen in this study, no significant differences were found in the PCV, WBC, RBC, Hb, MCV, MCH and MCHC in the BPH group relative to the control; similarly, no significant differences were found in the PCV, WBC, RBC, Hb, MCV, MCH and MCHC in the finasteride or test groups 1 and 2 rats relative to the BPH group, affirming the results of the acute toxicity study that showed the non-toxicity of the leaf extract of *S. malaccense*.

The histological findings of this study which revealed that the prostates of the finasteride and test groups 1 and 2 rats showed better prostate histology, and evidence of shrinkage of the prostate glands, unlike the BPH group prostate histology, reveal the promising potentials of *S. malaccense* in BPH management.

The presence of different antioxidant components in plants make it relatively difficult to measure each component separately (Eleazu et al., 2013). Given that antioxidants were defined as chemical compounds that have the capacity to quench free radicals (Halliwell and Gutteridge, 1998), in this study, the *in vitro* antioxidant activity of *S. malaccense *was quantified by measuring its scavenging activities on ABTS, hydrogen peroxide and nitric oxide radicals in comparison with the reference antioxidants, vitamin C for ABTS and hydrogen peroxide; and curcumin for nitric oxide. Although the IC_50 _values that were obtained for *S. malaccense *for the inhibition of these radicals were clearly higher than the reference antioxidants indicating lower antioxidant activity, nevertheless, the values that were obtained for *S. malaccense *indicate its strong antioxidant capacity. This finding was corroborated by the *in vivo* antioxidant activity of *S. malaccense *in the BPH animals. Our findings on the *in vitro* antioxidant capacity of *S. malaccense* lend credence to previous reports of Savitha et al. (2011), Batita et al. (2017) and Arumugam et al. (2014) on the strong *in vitro* antioxidant capacity of *S. malaccense *leaf*.*

HPLC characterization of methanol extract of the leaves of *S. malaccense* showed catechin, epicatechin and quercetin to be the bioactive constituents in it (Batista et al., 2017). These compounds were reported to possess antioxidant, anti-inflammatory and anti-proliferating properties (Arumugam et al., 2014; Ye et al., 2018).

It is therefore plausible to attribute the antioxidant, anti-inflammatory and BPH attenuating properties of *S. malaccense* as seen in this study to the presence of these polyphenol compounds in it. 

As far as we know, this is the first report on the usage of *S. malaccense* in mitigation of BPH. Whereas we were unable to determine the molecular basis of the BPH attenuating properties of *S. malaccense*, which we consider as a limitation in this study, a key strength of our study lies in the histology of the prostates of the BPH rats administered *S. malaccense *(at 100 and 200 mg/kg) that showed pictorial evidence of abrogation of BPH by *S. malaccense *as well as in the utility of this plant by humans, which makes it possible to replicate this study in human model of BPH. 

Our study reported for the first time, the beneficial use of *S. malaccense* **(**at 100 and 200 mg/kg) in the treatment of benign prostatic hyperplasia in rat model of BPH. The plant also modulated dyslipidaemia, hormonal alteration, systemic inflammation and oxidative stress in the BPH animals. The study therefore places spotlight on *S. malaccense* as a potential candidate for the treatment of benign prostatic hyperplasia. 
